# Postpartum depressive symptoms in the context of high social adversity and reproductive health threats: a population-based study

**DOI:** 10.1186/s13033-018-0219-x

**Published:** 2018-07-28

**Authors:** Telake Azale, Abebaw Fekadu, Charlotte Hanlon

**Affiliations:** 1 Department of Health Education and Behavioral Sciences, College of Medicine and Health Sciences, University of Gondar, Gondar, Ethiopia; 2 Centre for Global Mental Health, Institute of Psychiatry, Psychology and Neuroscience, King’s College London, London, UK; 3 Center for Innovative Drug Development and Therapeutic Trials for Africa, Addis Ababa University, Addis Ababa, Ethiopia; 4 Centre for Affective Disorders, Department of Psychological Medicine, Institute of Psychiatry, Psychology and Neuroscience, King’s College London, London, UK; 5 Department of Psychiatry, School of Medicine, College of Health Sciences, Addis Ababa University, Addis Ababa, Ethiopia

**Keywords:** Postpartum depression, Social determinants, Developing countries, Ethiopia

## Abstract

**Background:**

Postpartum depression is an important but neglected public health issue in low- and middle-income countries. The aim of this study was to assess postpartum depressive (PPD) symptoms and associated factors in a rural Ethiopian setting characterized by high social adversity and reproductive health threats. We hypothesized that infant gender preference would be associated with PPD symptoms.

**Methods:**

A cross-sectional, population-based study was conducted in Sodo district, southern Ethiopia, between March and June 2014. A total of 3147 postpartum women (one to 12 months after delivery) were recruited and interviewed in their homes. The questionnaire included demographic, reproductive health and psychosocial factors in addition to a culturally validated measure of depressive symptoms, the Patient Health Questionnaire. Scores of 5 or more were indicative of high levels of PPD symptoms.

**Results:**

The prevalence of high PPD symptoms was 12.2%, with 95% confidence interval (CI) between 11.1 and 13.4. Of these, 12.0% of the study participants had suicidal ideation. Preference of the husband for a boy baby was associated with PPD symptoms in univariate analysis (crude odds ratio 1.43: 95% CI 1.04, 1.91) but became non-significant after adjusting for confounders. In the final multivariable analysis, rural residence [adjusted odds ratio (aOR) 2.56: 95% CI 2.56, 4.19], grand multiparity (aOR 2.00: 1.22, 3.26), perinatal complications (aOR: 2.55: 1.89, 3.44), a past history of abortion (aOR 1.50: 1.07, 2.11), experiencing hunger in the preceding 1 month (aOR 2.38: 1.75, 3.23), lower perceived wealth (aOR 2.11: 1.19, 3.76), poor marital relationship (aOR 2.47: 1.79, 3.42), and one or more stressful events in the preceding 6 months (aOR 2.36: 1.82, 3.06) were associated significantly with high PPD symptoms.

**Conclusion:**

PPD symptoms affected more than one in 10 women in this Ethiopian community setting. Social adversity and reproductive health threats were associated with poorer mental health. Interventions focusing on poor rural women with low access to care are necessary. This research can serve as an entry point for the adaptation of a psychosocial intervention.

## Background

Postpartum depression (PPD) is a pressing but largely neglected public health concern in low- and middle-income countries (LMICs) [1]. The symptoms of PPD are the same as depressive symptoms at any other time in a person’s life [2]. The prevalence of PPD in LMICs is as high as, if not higher than, the prevalence seen in high-income countries [3]. Recent evidence on the prevalence of PPD was only available from 15% of LMICs [3] and indicated a weighted mean prevalence of perinatal common mental disorders (including depressive symptoms) of 19.8% [3]. In sub-Saharan Africa, many studies of PPD are facility-based or restricted to high risk populations, for example women with HIV, and are not representative of the general population of postpartum women. More representative community-based studies have mostly been conducted in Ethiopia and South Africa (Table 1). Most studies indicate a high burden of PPD symptoms, measured using validated depression screening scales, but with substantial variation in the estimated prevalence of PPD [4–7].

**Table 1:** Prevalence of postpartum depression in community-based studies from sub-Saharan Africa

Author, year	Country	Sample	Prevalence (%)	Measurement	Setting
Tsai et al. [76], (2016)	South Africa	1238	39.5	EPDS ≥ 13	Urban
Stellenberg et al. [6], (2015)	South Africa	159	50.3	EPDS ≥ 13	Rural
Hung et al. [77], (2014); Dewing et al. [68], (2013)	South Africa	249	31.7	EPDS ≥ 13	Urban
Cooper et al. [78], (1991)	South Africa	184	34.7	DSM-IV	Peri-urban
Ramchandani et al. [79], (2009)	South Africa	1035	16.0	PDQ ≥ 20	Urban
Tomlinson et al. [80], (2004)	South Africa	147	35.0	DSM-IV	Urban
Tefera et al. [4], (2015)	Ethiopia	340	31.5	SRQ ≥ 6	Urban
Baumgartner et al. [5], (2014)	Ethiopia	1319	32.8	SRQ ≥ 5	Rural and urban
Hanlon et al. [81], (2008)	Ethiopia	954	4.6	SRQ ≥ 6	Rural
Harpham et al. [82], (2005)	Ethiopia	1772	33	SRQ ≥ 6	Rural
Weobong et al. [7], (2015)	Ghana	13,360	3.8	PHQ ≥ 5	Rural

*DSM-IV*  Diagnostic and Statistical Manual of mental disorders fourth edition, *EPDS* Edinburgh Postnatal Depression Scale, *PDQ* Pitt Depression Questionnaire, *PHQ* Patient Health Questionnaire, *SCID* Structured Clinical Interview for DSM Disorders, *SRQ* Self-Reporting Questionnaire

PPD is likely to be an important factor contributing to elevated mortality in women in LMICs, through increasing the risk of suicide [8, 9]. There are several studies from LMICs which show a substantial burden of suicide in the perinatal period, for which unrecognized and untreated PPD is likely to have made an important contribution although information on the woman’s mental health prior to death is not known [10–12]. An estimated 20% of deaths among women in the postpartum period in India are classified as suicide or accidental burns [12]. In a hospital-based retrospective study from Mozambique, 33% of deaths during the early postpartum period were from suicide [10]. In Nepal, suicide was the leading cause of maternal death, accounting for 16% [11]. Similarly, 13% of maternal deaths were attributed to suicide in Sri Lanka [13]. PPD also has implications for the child and has been linked to higher rates of stunting, diarrheal diseases, lower completion of recommended schedules of immunization, lower rate of exclusive breast feeding and poorer cognitive and social-emotional development among children in LMICs [14–19].

Factors associated with PPD in LMICs include younger age [3, 20, 21], rural residence [3, 22], low income [3, 20, 23, 24], lack of social support [3, 24, 25], pregnancy and birth complications [3, 7, 22, 23, 26], unplanned pregnancy [3, 22, 27, 28], intimate partner violence [20, 22, 27] and non-adherence with perinatal sociocultural practices [29]. Gender preference has been frequently cited as a risk factor for PPD in Asian culture, but has not been adequately investigated in Africa [22, 23, 30–33]. In Ethiopia and other sub-Saharan African countries, studies indicate that gender preference affects the decision to use family planning [34, 35]. Couples have been found to postpone the use of family planning if their live children are female, reflecting a desire for a male child. In a previous qualitative study from Ethiopia, there was a strong preference for boy babies [36], but in a subsequent quantitative study no association was found between giving birth to a boy when the husband preferred a boy and onset of PPD, but this study was under-powered [37].

Inconsistencies in the prevalence of PPD and associated factors are likely to be explained by use of different measures of depressive symptoms and associated factors, differing cut-off scores on self-report measures of PPD, differing definitions of the perinatal period, variation in sample size, the nature of the sample (rural, periurban or urban) and differences in the variables included in multivariable models.

There is a need for rigorously conducted, contextually sensitive and adequately powered studies to investigate the distribution of perinatal depressive symptoms in rural sub-Saharan African country settings. The information obtained will help to inform appropriate intervention strategies at both the individual and community level. The aim of this study was to assess the prevalence of high levels of PPD symptoms and associated factors in a setting of high social adversity and reproductive health threats in Ethiopia. We tested the hypothesis that infant gender preference would be associated with PPD in rural Ethiopia.

## Methods

### Study design: a population-based cross-sectional survey

#### Study setting

The study was conducted in Sodo district, of the Gurage zone, Southern Nations, Nationalities and Peoples’ Region (SNNPR) of Ethiopia. SNNPR is one of the largest regions in Ethiopia, accounting for more than 10 percent of the country’s land area. The SNNPR is an extremely ethnically diverse region of Ethiopia. These ethnic groups are distinguished by different languages, cultures and socioeconomic organizations. The Gurage zone has 15 districts. Sodo district is the second largest in terms of population (161,952 persons; 79,356 men and 82,596 women), with 88% of the population residing in rural areas [38] and comprises 58 sub-districts. It is located about 100 km south of the capital city, Addis Ababa.

In Sodo district, there are eight health centers, each linked to five health posts served by health extension workers. There is a general hospital 30 km away from the district town, Buee, which has an outpatient psychiatric service provided by a psychiatric nurse. However, at the time of the study there was no specialist mental health professional located within the district and no health care personnel trained in mental health care. As part of the Program for Improving Mental health carE (PRIME), plans were being made to integrate mental health care into primary care and maternal health care settings across the district [39]. PRIME is a multi-country implementation research project involving five LMICs (Ethiopia, India, Nepal, South Africa and Uganda) [40]. The analyses presented in this paper were from a formative study which was conducted to identify the treatment gap for women with post-partum depression and their preferred help-seeking [41] and coping strategies [42] in order to inform service development. In this paper we focus on the identification of risk factors for development of PPD.

### Recruitment

We attempted to identify and recruit all women between one and 12 months postpartum with live infants who were residing in Sodo district. A total of 3147 women were recruited from the 58 sub-districts of the study district, identified by locators in a house-to-house census triangulated with the list of infants from the PRIME census [43] and immunization reports for the whole district obtained from the district health office. Further details of the sample identification have been described previously [41]. The eligibility criteria included being a resident of the study district for 1 year or more, having a live infant, being between one and 12 months postpartum and not exhibiting overt behavioral disturbance indicative of severe mental illness. Each household containing an eligible woman was visited by a data collector who then explained the purpose of the research and gave the woman an information sheet or read the information aloud for those who were unable to read. Women who consented to participate were interviewed at a time and place that was convenient for them, but for the most part the interview took place within their homes. The interviews took approximately 1 hour to complete.

### Measures

PPD symptoms were measured using the Patient Health Questionnaire (PHQ-9). The PHQ-9 was developed originally to measure depression in primary care settings [44]. The PHQ-9 has been culturally validated for use in several African country settings [3, 29, 45–47] including in postpartum women in rural Ghana [48] and in the primary health care and antenatal care settings in the neighbouring district to this study [49, 50]. In the Ethiopia primary care validation, a score of 5 or more was found to have a sensitivity of 83% and specificity of 75% for the detection of major depressive disorder. In antenatal women, the validated cut-off was four and above, giving a sensitivity of 86.7% and a specificity of 80.4%.

Gender preference was measured by asking the woman whether she was happy with her child’s gender (yes/no) and whether she perceived that her husband was happy with the child’s gender (yes/no).

#### Potential confounders/explanatory variables

Social support was assessed using the Oslo Social Support Scale (OSSS-3). The total score as well as the individual items of the OSSS-3 may be used. A total score ranging between 3 and 8 is classified as poor social support, a score between 9 and 11 as intermediate support, and a score between 12 and 14 as strong support [51]. The OSSS-3 has been used in Ethiopia in various settings, including the community for this study [43, 52–54].

Stressful events were measured by the list of threatening experiences (LTE-12) [55]. The LTE has been found to have convergent validity in various studies in Ethiopia [43, 53, 54]. Alcohol use disorder was indicated using the Fast Alcohol Screening Test [56], a four item questionnaire that has been adapted and used in the study site previously [43]. A score of 3 or more indicates probable hazardous or harmful drinking. Perinatal complications were assessed by asking the woman “Have you had pregnancy, or birth-related difficulties? If yes, what were they?” (coded as haemorrhage, prolonged labour or high blood pressure).

### Data collection and quality assurance

A total of 36 data collectors and four supervisors, who were recruited from the district by the PRIME project and had experience of data collection, were trained for 9 days. The educational levels of the data collectors ranged from tenth grade completed to first degree. They were supervised by four supervisors who were also trained and assisted by the investigators. The supervisors were diploma or degree graduates. A pre-test was conducted in three sub-districts near the study area. Data were collected between April and June 2014.

### Data management and analysis

Data were double entered into EpiData version 3.1 and exported to the Statistical Packages for Social Sciences, version 20 (SPSS-20) for analysis. Frequencies, percentages, and mean values were used to describe the categorical and continuous variables. Bivariate analyses were carried out to investigate the association between symptoms of PPD and several demographic, obstetric, and psychosocial variables. The hypothesis that the woman’s perception that her husband was unhappy with the gender of the baby would be associated with PPD was tested by controlling for demographic and obstetric factors in the multivariable analysis. All variables with a p-value < 0.2 were included in the multivariable model. Adjusted odds ratios with associated 95% confidence intervals were reported in the final multiple logistic regression model.

### Ethical considerations

Ethical approval was obtained from the Institutional Review Board of the College of Health Sciences, Addis Ababa University. Permission was also obtained from the Sodo District Health Office and administration. Women who agreed to participate gave written consent. For those who were not literate, independent witnesses were invited to sign to indicate that the information had been read out correctly. Non-literate participants then gave a finger print to indicate consent. Women who endorsed the PHQ item indicating suicidal ideation and those with higher than or equal to 10 in the PHQ were linked to the Butajira hospital psychiatric nurse-led outpatient clinic.

## Results

A total of 3147 women between one and 12 months postpartum (mean 5.89 months postpartum; standard deviation (SD) 3.42) were included in the study. One woman was excluded and referred for specialist mental health care with probable psychotic symptoms. No women refused to participate in the study.

The mean age of the respondents was 27.9 years (SD 5.3). Concerning the gender of the baby, 8.8% (n = 276) of the women and 11.6% (n = 366) of their husbands reported being unhappy. More husbands were happy about the baby’s gender if the baby was male (55.9% for male vs. 44.1% for female) (Table 2).

**Table 2:** Socio-demographic, obstetric, and psychosocial characteristics of postpartum women in Sodo district, Ethiopia (n = 3147)

Characteristics of participants	Frequency	Percent
Age (years)		
Less than 20	107	3.4
20–29	1754	55.7
30–39	1217	38.7
40 or more	69	2.2
Marital status		
Not currently married	51	1.6
Married	3096	98.4
Residence		
Rural	2788	88.6
Urban	359	11.4
Education attended		
No formal education	2212	70.3
Primary education	802	25.5
Secondary and above	133	4.2
Occupational status		
Housewife	2722	86.5
Government or private employee	351	11.2
No job or daily laborer	74	2.4
Religion		
Orthodox Christian	2931	93.1
Protestant, Muslim, Catholic or other	216	6.9
Ethnicity		
Gurage	2856	90.8
Amhara, oromo or other	291	9.2
Baby gender		
Male	1607	51.1
Female	1540	48.9
Happy about baby gender		
Yes	2871	91.2
No	276	8.8
Husband happy about baby gender		
Yes	2781	88.4
No	366	11.6
Perinatal complication^a^		
Yes	346	11.0
No	2801	89.0
History of stillbirth		
Yes	162	5.1
No	2985	94.9
History of abortion		
Yes	296	9.4
No	2851	90.6
Parity of women		
Primipara	504	16.0
Multipara	1393	44.3
Grand multipara	1250	39.7
Experienced hunger in the past 1 month		
Yes	434	13.8
No	2713	86.2
Relative wealth		
Less	800	25.4
Same	2083	66.2
Better	264	8.4
Level of social support		
Poor support	569	18.1
Intermediate support	1565	49.7
Strong support	1013	32.2
Stressful event in the past 6 months		
No	1621	51.5
1 stressful event	715	22.7
2 or more stressful events	811	25.8
Husband drinks too much alcohol		
No	2450	77.9
Yes	697	22.1
Marital relationship		
Poor	291	9.2
Good	2856	90.8
Problem drinking (≥ 3 on the FAST)		
No	3121	99.2
Yes	26	0.8

*FAST* Fast Alcohol Screening Test

^a^Bleeding in pregnancy or post-partum period, prolonged labour or high blood pressure

The prevalence of high PPD symptoms (PHQ-9 score of 5 or more) was 12.2% (385/3147) with 95% confidence interval 11.1–13.4. The prevalence estimates for PPD symptoms did not differ across the postpartum period: 1–3 months postpartum 12.7% (129/1012), 4–6 months 11.1% (80/723) and 7–12 months 12.5% (176/1412).

### Factors associated with PPD symptoms

In the bivariate analysis, the odds of having PPD symptoms were 1.43 times higher in women whose husbands were not happy about the baby gender: 95% confidence interval (CI) 1.04–1.91. However, the association became non-significant in the multivariable model. In the multivariable analysis, the following were associated significantly with PPD symptoms: rural residence, grand multi-parity, history of complication during pregnancy of the index child, past history of abortion, experiencing hunger in the preceding month due to lack of food, perceived wealth less than the neighbors, poor marital relationship, and having had one or more negative events during the preceding 6 months (Table 3).

**Table 3:** Crude and adjusted odds ratios for factors associated with postpartum depressive symptoms in women from Sodo district, Ethiopia

Characteristic	Postpartum depressive symptoms (Patient Health Questionnaire-9 ≥ 5) N = 3147		Crude odds ratio (95% confidence interval)	Adjusted Odds ratio (95% confidence interval) (n = 3147)
	Yes 385 (12.2)	No 2762 (88.8)		
Age (years)				
< 20	11 (10.3)	96 (89.7)	Reference	Reference
20–29	191 (10.9)	1563 (89.1)	1.06 (0.56, 2.02)	2.38 (0.76, 7.39)
30–39	175 (14.4)	1042 (85.6)	1.46 (0.77, 2.79)	1.90 (0.82, 4.38)
40 or more	8 (11.6)	61 (88.4)	1.14 (0.43, 3.00)	1.87 (0.83, 4.20)
Not currently married	11 (21.6)	40 (78.4)	*2.00 (1.01, 3.93)*	1.05 (0.46, 2.40)
Rural residence	363 (13.0)	2425 (87.0)	*2.93 (1.46, 3.57)*	*2.60 (1.58, 4.27)*
No formal education	308 (13.9)	1904 (86.1)	*1.80 (1.38, 2.34)*	1.31 (0.96, 1.80)
Husband unhappy about baby gender	58 (15.8)	308 (84.2)	*1.43 (1.04, 1.91)*	1.20 (0.77, 1.85)
Perinatal complication	86 (24.9)	260 (75.1)	*2.76 (2.10, 3.63)*	*2.43 (1.80, 3.29)*
History of stillbirth	37 (22.8)	125 (77.2)	*2.24 (1.52, 3.29)*	1.42 (0.92, 2.19)
History of abortion	61 (20.6)	235 (79.4)	*2.02 (1.49, 2.74)*	*1.45 (1.03, 2.04)*
Parity				
Primiparous	40 (7.9)	464 (92.1)	*Reference*	*Reference*
2–4 live births	149 (10.7)	1244 (89.3)	1.38 (0.96, 2.00)	1.43 (0.91, 2.23)
Five or more	196 (15.7)	1054 (84.3)	*2.15 (1.50, 3.08)*	*1.94 (1.18, 3.20)*
Hunger in the previous month	126 (29.0)	308 (71.0)	*3.87 (3.03, 4.94)*	*1.95 (1.43, 2.67)*
Relative wealth				
Less	171 (21.4)	629 (78.6)	*4.21 (2.47, 7.18)*	*1.84 (1.03, 3.29)*
Same	198 (9.5)	1885 (90.5)	*1.62 (0.96, 2.75)*	*1.34 (0.77, 2.31)*
Better	16 (6.1)	248 (93.9)	*Reference*	*Reference*
Social support				
Poor	104 (18.3)	465 (81.7)	*2.18 (1.61, 2.95)*	0.89 (0.66, 1.19)
Intermediate	187 (11.9)	1378 (88.1)	*1.32 (1.02, 1.72)*	0.81 (0.58, 1.14)
Strong	94 (9.3)	919 (90.7)	*Reference*	*Reference*
Husband does not live in the same house	70 (18.0)	319 (82.0)	*1.70 (1.28, 2.26)*	1.31 (0.93, 1.83)
Husband drinks too much alcohol	95 (13.6)	602 (86.4)	1.17 (0.91, 1.50)	0.79 (0.60, 1.04)
Poor marital relationship	80 (27.5)	211 (72.5)	*3.17 (2.38, 4.21)*	*2.13 (1.53, 2.97)*

Reference category

The variables with statistically significant association (p < 0.05) are written in italics

## Discussion

In this paper we report findings from a large population-based study of PPD symptoms in a rural Ethiopian setting, using a culturally validated measure of depression and a wide range of potentially relevant associated factors measured using standardized instruments. The hypothesis that unhappiness of the father about the baby’s gender would be associated with PPD symptoms was rejected.

The prevalence of high PPD symptoms in our study was 12.2%. Although this is in the range of the prevalence reports from community studies from LMICs (ranging from 4.9 to 59.4%) [3], it is much higher than a previous study conducted in a neighboring district 10 years ago where the prevalence was 5% [57]. Apart from the difference in the measurement instruments (the Self-Reporting Questionnaire was used in the previous study), it is possible that psychosocial and socio-cultural protective factors might be declining in the society. Socio-cultural practices that provide emotional and material support to women following birth are hypothesized to be protective against PPD or may be risk factors when people fail to comply with them [36].

In the same sample of women, we have shown previously that the treatment gap for PPD is very high [41], with most women not seeking any help for their symptoms and not receiving any evidence-based care. Nonetheless, around half of women were receptive to receiving treatment in the primary healthcare setting as well as from religious to traditional healers.

### Gender preference, social adversity and PPD

Symptoms of PPD were higher among women whose husbands were not happy about the baby gender (15.8% vs. 11.8%), p-value < 0.05. However, this association became non-significant after adjusting for potential confounders. Gender preference has been reported as independent predictor of PPD in Asian countries [33, 58–61]. In most of the studies conducted in LMICs, male gender was preferred to female especially among people with low income and education [58, 62, 63]. Although there is some evidence of gender preference in the Ethiopian setting [34], this does not appear to translate into a threat to the mental health of perinatal women.

The association between depression and disadvantage in women, including gender inequality, intimate partner violence and low maternal education, has been reported by many studies in LMICs, including Ethiopia [64, 65]. In this study, women living in the rural area had about twice the odds of having PPD. Rural living is associated with lower socioeconomic status, lower empowerment of women and poorer access to healthcare in Ethiopia. Unemployment and poverty are well known risk factors for PPD [3, 20, 23, 24, 66, 67]. Dimensions of poverty include food and financial insecurity. In this study, women who had experienced hunger in the preceding month and who perceived their socio-economic status to be lower than others were more likely to have PPD. This is consistent with findings in other LMICs [66, 68, 69]. Those women who reported a poor relationship with their husband were also more likely to have PPD. The question of whether poor marital relationships cause PPD or PPD leads to problems in the marital relationship remains unanswered given the cross-sectional nature of our study. Nearly half of women in our study had experienced at least one stressful life event, ranging from loss of a loved one to being the victim of theft, and this was associated significantly with PPD.

The importance of social determinants of PPD in this rural Ethiopian setting is also reflected in our previous finding of attribution of PPD symptoms to social rather than psychological causes [36, 41]. Any intervention for PPD will need to consider social determinants in order to effectively address the underlying cause of depression, as well as to be acceptable for women in this context. Poverty reduction interventions or interventions to address intimate partner violence would be expected to improve mental health in women in this setting at the population level, although a review indicated that micro-finance initiatives may actually increase mental distress [70]. Nonetheless, alongside such initiatives, it is likely that individually-focused psychosocial interventions will also be needed for a sub-group of women. In a systematic review of psychosocial interventions for women with perinatal depression, there was preliminary evidence that purely social interventions were less effective than psychological interventions [71].

### Reproductive health threats

Women who had given birth to five or more children had two-fold increased odds of experiencing PPD compared to first time mothers. This is in keeping with previous studies from LMICs [3, 69, 72]. The unmet need for family planning is very high in many LMICs and women with high fertility are more likely to be uneducated, poor and in poorer health, all of which are associated with PPD [73]. Past adverse pregnancy outcomes such as abortion and perinatal complications were found to have significant association with PPD in this study, as with many other studies in other LMICs [3, 7, 22, 23, 26, 69]. Despite recent reductions, maternal mortality remains high in rural Ethiopian settings. As a consequence, any medical complications during pregnancy are likely to be perceived as potentially life-threatening and a potent threat to mental health [74]. Loss of a previous pregnancy has been associated with increased risk of mental health problems in the subsequent pregnancy in high-income country settings [75] but has been little-investigated in LMICs. Improving the reproductive health of women would be expected to improve their mental as well as their physical health. Nonetheless, for women who do experience complications, improved psychological support may reduce the risk of developing future mental health problems.

### Limitations

This is a cross-sectional study it is difficult to determine temporal relationship between exposure and outcome variables, for example between PPD and poverty. Reliance on self-report for the measurement of factors such as wealth, marital relationship and husbands’ substance use may have led to under-reporting of the true extent of the problem. Although women were asked specifically about symptoms for PPD in the preceding 2 weeks, some might have also reported symptoms present during or before pregnancy due to difficulty defining the recall period in this rural setting with low levels of literacy. Some depressive symptoms, such as weakness, may have been the result of the demands of the postpartum period rather than depression. We did not conduct physical examinations and may have missed underlying physical health problems.

## Conclusion

Postpartum depression affected at least one in ten women in this Ethiopian community. Social adversity and reproductive threats were high and associated with PPD. Improving reproductive healthcare, addressing social determinants of PPD and creating access to mental health care through integration into existing primary care-based maternal health care may reduce the burden.

## Abbreviations

DSM-IV: Diagnostic and Statistical Manual of mental disorders fourth edition
EPDS: Edinburgh Postnatal Depression Scale
LMIC: low- and middle-income country
LTE: list of threatening experiences
OSSS: Oslo Social Support Scale
PDQ: Pitt Depression Questionnaire
PHQ: Patient Health Questionnaire
PRIME: Programme for Improving Mental health carE
SCID: Structured Clinical Interview for DSM Disorders
SNNPR: Southern Nations, Nationalities and People’s Region
SRQ: Self-Reporting Questionnaire
